# Diversity of Antimicrobial Resistance Phenotypes in *Salmonella* Isolated from Commercial Poultry Farms

**DOI:** 10.3389/fvets.2017.00096

**Published:** 2017-06-23

**Authors:** Karen A. Liljebjelke, Charles L. Hofacre, David G. White, Sherry Ayers, Margie D. Lee, John J. Maurer

**Affiliations:** ^1^Department of Population Health, College of Veterinary Medicine, Athens, GA, United States; ^2^Center for Veterinary Medicine, U.S. Food and Drug Administration, Laurel, MD, United States

**Keywords:** *Salmonella*, strain type, antimicrobial resistance, poultry, vertical transmission

## Abstract

*Salmonella* remains the leading cause of foodborne illness in the United States, and the dissemination of drug-resistant *Salmonellae* through the food chain has important implications for treatment failure of salmonellosis. We investigated the ecology of *Salmonella* in integrated broiler production in order to understand the flow of antibiotic susceptible and resistant strains within this system. Data were analyzed from a retrospective study focused on antimicrobial resistant *Salmonella* recovered from commercial broiler chicken farms conducted during the initial years of the US FDA’s foray into retail meat surveillance by the National Antimicrobial Resistance Monitoring System (NARMS). Sixty-three percentage of *Salmonella* were pan-susceptible to a panel of 19 antimicrobials used by the NARMS program. Twenty-five antimicrobial resistance phenotypes were observed in *Salmonella* isolated from two broiler chicken farms. However, *Salmonella* displaying resistance to streptomycin, alone, and in combination with other antibiotics was the most prevalent (36.3%) antimicrobial resistance phenotype observed. Resistance to streptomycin and sulfadimethoxine appeared to be linked to the transposon, Tn*21*. Combinations of resistance against streptomycin, gentamicin, sulfadimethoxine, trimethoprim, and tetracycline were observed for a variety of *Salmonella enterica* serovars and genetic types as defined by pulsed-field gel electrophoresis. There were within and between farm differences in the antibiotic susceptibilities of *Salmonella* and some of these differences were linked to specific serovars. However, farm differences were not linked to antibiotic usage. Analysis of the temporal and spatial distribution of the endemic *Salmonella* serovars on these farms suggests that preventing vertical transmission of antibiotic-resistant *Salmonella* would reduce carcass contamination with antibiotic-resistant *Salmonella* and subsequently human risk exposure.

## Introduction

*Salmonella* remains the leading cause of outbreak-associated gastroenteritis in the United States, and consumption of poultry products has been implicated in several of these outbreaks (1, 2). Since implementation of the HACCP program, improvement has been made in the level of *Salmonella* contamination of processed chicken carcasses (3). However, a survey of retail meat from the Washington, DC, USA area revealed a surprising level of contamination of beef, pork, and poultry products with antibiotic-resistant *Salmonella* (4, 5). The dissemination of antibiotic-resistant *Salmonella* through the food chain has important public health implications considering the potential for treatment failure when cases of gastroenteritis require medical intervention, especially in children, the elderly, and the immunocompromised (6). In addition, infections with antimicrobial resistant bacteria including *Salmonella* have been associated with higher rates of morbidity and mortality (7–9).

The use of antibiotics in food animal production has been implicated as a contributing factor to the emergence of drug resistance in human foodborne pathogens (6, 10). The emergence and rapid worldwide spread of the multiple drug-resistant *Salmonella enterica* Typhimurium phage-type DT104 clone and ceftriaxone-resistant *S. enterica* serovars Heidelberg, Newport, and Typhimurium have underscored the threat to both animal agriculture and human health posed by multiple drug-resistant pathogens (11–15). Antimicrobial resistance genes are widely disseminated in pathogenic, commensal, and environmental bacteria (16, 17). Furthermore, it has been shown that once antimicrobial resistance has been introduced into an ecosystem, resistance can spread and persist without continuing selection pressure from antibiotics (18, 19). In addition, the reservoir of antimicrobial resistance genes is larger than previously thought (20). It is in this environment that the potential exists for *Salmonella* to acquire antimicrobial resistance genes from resident poultry microbiota due to selection pressure from therapeutic and non-therapeutic antibiotic usage. It follows then that the longer *Salmonellae* persists in the environment of an animal production facility, the chance of acquiring resistance genes increases.

We took advantage of the integrated nature of poultry production to observe the antimicrobial resistance phenotypes acquired by salmonellae during broiler chicken production in order to identify potential critical control points for *Salmonella* contamination and antimicrobial resistance development; ultimately in order to provide information relevant to reducing the level of carcass contamination with antibiotic-resistant *Salmonella*. Data were analyzed from a retrospective study focused on antimicrobial-resistant *Salmonella* recovered from commercial broiler chicken farms conducted during the initial years of the US FDA’s foray into National Antimicrobial Resistance Monitoring System (NARMS) retail meat surveillance (4). Despite the diversity of antimicrobial resistance profiles, poultry *Salmonella* recovered from these farms in 2003 were generally susceptible to the tested antimicrobials of animal and human health significance. Vertical transmission appeared to be the most important factor in chicken carcass contamination with antibiotic-resistant *Salmonella*.

## Materials and Methods

### Description of Antimicrobial Usage for Two Commercial Broiler Chicken Farms in Northeast Georgia

Selection and description of study farms was as previously described (21). Approximately 17,000 chicks were placed in each house on Farm One. No litter amendments were used (22). At the hatchery, gentamicin was administered *in ovo* (0.1 mg/egg) on day 17 of development. No antibiotics were used therapeutically on this farm to treat birds during this study. Chicks were fed starter feed containing virginiamycin (10 g/ton) (25 g/ton) for the first 2 weeks. The starter feed contained coccidiostat rotated in the following order: Flock 1; diclazuril (1 g/ton), Flock 2; narasin (72 g/ton), Flock 3; monensin (100 g/ton), Flocks 4, 5; nicarbazin (82 g/ton), and Flocks 6, 7; salinomycin (60 g/ton). Flocks were fed grower feed for the next 2 weeks containing bacitracin (25 g/ton), and other coccidiostats rotated in the following order: Flock 1; salinomycin (60 g/ton), Flocks 2, 3; narasin (72 g/ton), Flocks 4, 5; lasalocid (82 g/ton), and Flocks 6, 7; diclazuril (1 g/ton). Finisher feed containing virginiamycin (15 g/ton), without coccidiostat was fed for 1–2 weeks as birds approached market weight. Withdrawal feed containing neither antibiotics nor coccidiostats was fed for the last week of grow-out. Feed was withdrawn for 16 h prior to catch.

Approximately 20,000 chicks were placed per house on Farm Two. No litter amendments were used on Farm Two (22). At the hatchery, gentamicin (0.2 mg/chick) was injected subcutaneously into day-of-hatch chicks. Chicks were reared on starter feed containing bacitracin (25 g/ton), and salinomycin (50 g/ton) for the first 2 weeks, then grower feed containing bacitracin (25 g/ton) and salinomycin (50 g/ton) for 2 weeks, then finisher feed without growth promotant or coccidiostat for 1–2 weeks. Withdrawal feed without antibiotic or coccidiostat was fed for the last week of grow-out. Feed was withdrawn for 16 h prior to shipment. *Escherichia coli* airsacculitis was diagnosed in house B during week six of Flock 3 on Farm Two, and oxytetracycline was administered in drinking water at 10.4 mg/kg weight for 1 day and at 5.1 mg/kg weight for 4 days. In this work, we sampled chick box liners, the poultry environment, and chicken carcasses. The latter was provided to us by the participating poultry companies. We did not physically interact with chickens raised on these farms and, therefore, we were exempt from university guidelines and USDA/NIH regulations regarding animal use.

### Genotypic and Phenotypic Characterization of Poultry *Salmonella* Isolates

The 289 *Salmonella* strains, examined in this study, were isolated, serotyped, phage-typed, and strain-typed as previously described (21). Presence of *aadA1* and *merA* was determined as described by Bass et al. (23).

Antibiotic susceptibility was determined for the 289 archived *Salmonella* isolates (21). The minimum inhibitory concentration (MIC) of the antimicrobial agents tested was determined with the Sensititre^®^ automated antimicrobial susceptibility system (Trek Diagnostic Systems, Westlake, OH, USA) and interpreted according to the National Committee for Clinical Laboratory Standards (NCCLS) guidelines for microbroth dilution methods (24, 25). Sensititre^®^ susceptibility testing was performed according to the manufacturer’s instructions, and susceptibility and resistance were reported as MIC (μg/ml). Three-letter abbreviations and resistance breakpoint concentration are in parentheses. The antimicrobials assayed were as follows: amikacin (AMI > 64 μg/ml), amoxicillin/clavulanic acid (AUG > 32/16 μg/ml), ampicillin (AMP > 32 μg/ml), apramycin (APR 32 µg/ml), ceftriaxone (AXO > 64 μg/ml), cefazolin (CEF 32 µg/ml), cefoxitin (FOX > 32 μg/ml), ceftiofur (TIO > 8 μg/ml), cephalothin (CEP > 32 μg/ml), chloramphenicol (CHL > 32 μg/ml), ciprofloxacin (CIP > 4 μg/ml), kanamycin (KAN 64 µg/ml), gentamicin (GEN > 16 μg/ml), imipenem (IMP > 4 μg/ml), nalidixic acid (NAL > 32 μg/ml), streptomycin (STR > 64 μg/ml), sulfadimethoxine (SMX > 512 μg/ml), tetracycline (TET > 16 μg/ml), and trimethoprim/sulfamethoxazole (TMS > 4/76 μg/ml). The antibiotics bacitracin and virginiamycin were not included with this panel as there is no breakpoint for *Salmonella* as their activity is specifically directed toward Gram-positive bacteria and it is used to prevent *Clostridium perfringens* infections in chickens.

This study was performed in 2003, early in the US Food and Drug Administration’s survey of antimicrobial-resistant foodborne bacteria recovered from retail meats, using the same methods and antimicrobial resistance break points recommended by NCCLS (Clinical and Laboratory Standards Institute) at that time.

### Statistical Analysis

The Fisher’s exact test with α = 0.05 and Mantel–Haenszel chi-squared test were used to test for non-random associations between specific data values. *Salmonella* Typhimurium PFGE types 1.1, 1.2, and 1.3 were ranked with regard to multiple drug resistance as determined by fitting linear model: log (μ_i_) = β_o_ = β_1_*PFGE type_i_, μ_i_ = mean number antimicrobial resistances or resistance type, with assumption that data conformed to Poisson distribution.

## Results

### Antibiotic Susceptibility and Diversity of Antimicrobial Resistance Phenotypes in Poultry *Salmonella*

There is ample opportunity for antibiotic-resistant *Salmonella* to emerge on poultry farms due to the combination of on farm antibiotic usage and the significant reservoir of antimicrobial resistance genes present in poultry litter. We examined the antibiotic susceptibility of *Salmonella* collected from two commercial broiler farms in northeast Georgia in relation to on-farm antibiotic usage. The majority of *Salmonella* isolates (62.6%; *n* = 172) were susceptible to all 19 antimicrobials tested, with the remainder displaying resistance to streptomycin (30.9%), gentamicin (12.6%), sulfadimethoxine (20.9%), tetracycline (13.9%), and trimethoprim/sulfamethoxazole (8.6%) (Table 1). *Salmonella* resistance to streptomycin alone was the most prevalent antimicrobial resistance phenotype (30.9%) (Tables 1 and 2).

**Table 1:** Most prevalent antimicrobial resistance phenotypes observed in the *Salmonella* serovars isolated from production and processing of seven consecutive commercial broiler flocks.

*Salmonella enterica* serovar (*n*=)	% Sensitive^a^	% STR^a^	% GEN^a^	% SMX^a^	% TET^a^	% TMS^a^	% AMP^a^	% Multidrug resistant^b^
**Farm One**								
*S*. Typhimurium (153)	66.6^d^	36.6	9.8	12.4	5.9	1.9	0.6	11.1
*S*. Enteritidis (28)	92.8^d^	3.6	3.6	3.6	0	0	3.6	3.6
*S*. Montevideo (22)	40.9	18.2	0	54.5	59.1	59.1	0	53.8
*S*. Kentucky (13)	23.1^c^	61.5	61.5	76.9	7.7	7.7	0	53.8
*S*. Heidelberg (6)	33.3	50.0	33.3	33.3	16.7	16.7	16.7	66.7
All isolates (241)	60.7	35.4^c^	13.8	23.4^c^	13.3	10.4^c^	0.9	22.7
**Farm Two**								
*S*. Kentucky (13)	100^c^	0	0	0	0	0	0	0
*S*. Mbandaka (9)	55.6	11.1	11.1	11.1	33.3	0	0	11.1
*S*. Typhimurium (6)	66.6	33.3	16.7	16.7	16.7	0	0	16.7
*S*. Ohio (5)	80.0	20.0	0	0	0	0	0	0
*S*. Senftenberg (4)	75.0	0	25.0	25.0	25.0	0	25.0	25.0
All isolates (48)	72.3	8.5^c^	6.4	8.5^c^	17.0	0^c^	4.3	10.6

*^a^Resistance profiles to the following antibiotics: AMP, ampicillin; GEN, gentamicin; STR, streptomycin; TET, tetracycline; SMX, sulfadimethoxine; and TMS, trimethoprim/sulfamethoxazole. Sensitive: susceptible to the 19 antibiotics tested*.

*^b^Resistance to three or more antibiotics*.

*^c^Farm differences in isolate or serovar susceptibility to antibiotics as determined by chi-squared test (*p* < 0.05)*.

*^d^*Salmonella* serovar differences in susceptibility to antibiotics as determined by chi-squared test (*p* < 0.05)*.

**Table 2:** Diversity of antimicrobial resistance phenotypes in *Salmonella* isolated from two commercial poultry farms.

Antimicrobial resistance phenotypes^a^	Strain type^b^	Total^c^		
STR	5	41	(36.28)	
STR SMX GEN	5	17	(15.04)	
SMX TMS TET	4	11	(9.73)	
STR SMX GEN TET	3	8	(7.08)	
STR SMX TMS TET	5	7	(6.19)	
STR SMX	2	3	(2.65)	
STR SMX GEN TMS TET	2	3	(2.65)	
CHL	2	2	(1.77)	
STR SMX GEN CEP	1	2	(1.77)	
STR TET CHL	1	2	(1.77)	
TET	2	2	(1.77)	
TET CEP CHL	2	2	(1.77)	
AMP	1	1	(0.88)	
STR SMX CEP	1	1	(0.88)	
STR SMX AXO FOX TIO AMI APR NAL	1	1	(0.88)	
STR SMX GEN AMP	1	1	(0.88)	
SMX GEN	1	1	(0.88)	
STR GEN	1	1	(0.88)	
STR SMX GEN TET AMP	1	1	(0.88)	
SMX TET TMS CEP CHL KAN	1	1	(0.88)	
STR SMX TET TMS CHL	1	1	(0.88)	
CEP AMP	1	1	(0.88)	
SMX TMS	1	1	(0.88)	
TMS TET	1	1	(0.88)	
SMX	1	1	(0.88)	
“STR SMX” alone or with another antimicrobial resistance		47	(41.59)	*p* < 0.05^d^
“STR SMX GEN” alone or with another antimicrobial resistance		32	28.32%	*p* < 0.05^e^
Diversity (Reciprocal Simpson’s Index) = 1.20				
Evenness = 0.26				

*^a^Resistance profiles to the following antibiotics: AMP, ampicillin; AUG, augmentin; FOX, cefoxitin; CEP, cephalothin; GEN, gentamicin; KAN, kanamycin, STR, streptomycin; AMI, amikacin; NAL, nalidixic acid; TET, tetracycline; SMX, sulfadimethoxine; TMS, trimethoprim/sulfamethoxazole; and CHL, chloramphenicol*.

*^b^Number of different S. enterica serovar or strain type with antimicrobial resistance phenotype*.

*^c^( ) Percentage of total antimicrobial resistance phenotypes identified*.

*^d^Linkage of STR with SMX as determined by the chi-squared test*.

*^e^Linkage between STR and SMX with GEN as determined by the chi-squared test*.

A diversity of antimicrobial resistance phenotypes (*n* = 25) was observed among the *Salmonella* isolated from commercial broiler chicken farms (Table 2). Twenty percentage of our poultry *Salmonella* isolates were resistant to three or more antibiotics (Table 1). The most common antimicrobial resistance phenotypes identified were to streptomycin (36.28%); streptomycin and sulfadimethoxine, alone or in combination with other antibiotics (41.59%); and streptomycin, sulfadimethoxine, and gentamicin, alone or in combination with other antibiotics (28.32%) (Table 2). There was a statistically significant association between *Salmonella* isolates displaying resistance to streptomycin and sulfadimethoxine; and streptomycin, sulfadimethoxine, and gentamicin (chi-squared test: *p* < 0.05). While antimicrobial resistance phenotype diversity was high (Reciprocal Simpson’s Index: 1.20), evenness in distribution of these phenotypes among *Salmonella* was low (0.26). The low evenness score may be a reflection of the broad distribution of certain antimicrobial resistance phenotypes compared to others [streptomycin resistance, alone (41 strain types); streptomycin, sulfadimethoxine, and gentamicin resistance (17 strain types); sulfadimethoxine, trimethoprim/sulfamethoxazole, and tetracycline resistance (11 strain types); streptomycin, sulfadimethoxine, gentamicin, and tetracycline resistance (8 strain types); streptomycin, sulfadimethoxine trimethoprim/sulfamethoxazole, and tetracycline resistance (7 strain types)].

Two of the common antimicrobial resistances identified, streptomycin and sulfadimethoxine resistance, are commonly associated with the transposon, Tn*21*. The resistance genes *merA* and *aadA1* are resident on this mobile genetic element and the distribution of these loci was 17.86 and 10.56%, respectively, in the recovered poultry isolates. There was a significant association between these resistance genes and resistance to streptomycin or sulfadimethoxine (chi-squared test; *p* < 0.05).

### Farm Variability in Antimicrobial Susceptibilities of Poultry *Salmonella*

Differences were observed within and between poultry farms in antibiotic susceptibilities of *Salmonella* isolates. Antimicrobial susceptibility patterns differed between farms as *Salmonella* isolates from Farm One were more likely to be resistant to streptomycin, sulfadimethoxine, and trimethoprim/sulfamethoxazole compared to those recovered from Farm Two (chi-squared test: *p* < 0.05) (Table 1). There were also differences in antibiotic susceptibilities among certain *Salmonella* serovars within farms as well as between farms. *S*. Typhimurium isolated from Farm One were less susceptible to antibiotics, tested in this study, than *S*. Enteritidis isolated from the same farm. *Salmonella* Kentucky isolated from Farm One exhibited significantly more antimicrobial resistance than other *Salmonella* isolated from the same farm as well as *S*. Kentucky isolated from Farm Two (Table 1). Following tetracycline treatment on Farm Two, *Salmonella* isolates were less likely to be resistant to tetracycline, as determined using one-sided, Fisher’s exact test at α = 0.05 (*p* = 0.0046), or to other antibiotics (Cochran–Mantel–Haenszel method, *p* = 0.0046). The therapeutic treatment of *E. coli* airsacculitis with tetracycline did not seem to selectively enrich for antimicrobial resistance in *Salmonella* isolated from subsequent flocks. In addition, there was no statistically significant difference in *Salmonella* isolates displaying resistance to tetracycline between the two poultry farms (chi-squared test; *p* = 0.34).

### Is Horizontal or Vertical Transmission Responsible for Spread of Antibiotic-Resistant *Salmonella* to Poultry Meat?

*S*. Typhimurium (*n* = 159) was the most prevalent serovar isolated in this study, and this serovar was frequently isolated from Farm One. Serovar Typhimurium isolates were largely pan-susceptible (66.6%); however, the most prevalent antimicrobial resistance phenotypes were to streptomycin (6.6%), sulfadimethoxine (12.4%), gentamicin (9.4%), and tetracycline (6.4%) (Table 3). Resistance to the other 14 antimicrobials tested was not observed that often (≤5%). Eleven percentage of *S*. Typhimurium isolates were resistant to three or more antibiotics. The most prevalent *S*. Typhimurium resistance phenotypes observed were as follows: streptomycin alone (23.7%) and the multi-drug resistant phenotype to streptomycin, gentamicin, sulfadimethoxine, and tetracycline (5.3%). A diversity of antimicrobial resistance phenotypes (*n* = 9) was observed for the three related *S*. Typhimurium strain types identified by PFGE (Table 3). Combinations of resistance against streptomycin, gentamicin, sulfadimethoxine, and tetracycline, accounted for 85.3% of the resistance phenotypes (Table 3). There was no significant difference in resistance phenotypes between the three *S*. Typhimurium genetic types isolated from Farm One with the exception that PFGE type T1.3 was significantly more likely to be ampicillin resistant (α = 0.05).

**Table 3:** Antimicrobial resistance phenotypes of *Salmonella enterica* serovars and strain types isolated from commercial broiler chicken farms.

*Salmonella* serovar (phage type)^a^^,^^b^	PFGE type^b^	Antimicrobial resistance phenotype^c^	Number of isolates
*S*. Enteritidis (PT8)	E1.1	Sensitive	18
		AMP	1
		STR SMX GEN	1
	E1.2	Sensitive	8

*S*. Typhimurium (DT193)	T1.1	Sensitive	50
		STR	16
		STR SMX	1
		STR SMX GEN TET	3
		STR SMX TET TMS	1
		STR SMX GEN TET TMS	2

(DT107)	T1.2	Sensitive	47
		STR	21
		STR SMX CEP	1
		STR SMX GEN	5
		STR SMX GEN TET	3
		STR SMX AXO FOX TIO AMI APR NAL	1

(U302)	T1.3	Sensitive	5
		STR GEN SMX AMP	1

(NT)	T2	Sensitive	1
	T3	Sensitive	1

*S*. Montevideo	V1.1	Sensitive	6
	V1.2	SMX TET TMS	3
	V1.3	SMX TET TMS	1
	V1.5	SMX TET TMS	6
		STR SMX TET TMS	3
	NT	Sensitive	1
		STR	1
		CHL	1

*S*. Kentucky	NT	Sensitive	16
		STR SMX	2
		GEN SMX	1
		STR SMX GEN	6
		STR SMX GEN TET TMS	1

*S*. Senftenberg	S1	Sensitive	3
		STR GEN	1
		STR SMX GEN	4
		STR SMX GEN TET AMP	1
		STR SMX GEN CEP	2

*S*. Gaminara	G1.1	SMX TET TMS CEP CHL KAN	1
		STR TET CHL	2
		STR SMX TET TMS CHL	1
	G1.2	Sensitive	2
	G2.1	CEP AMP	1
	G3.1	Sensitive	1

*S*. Mbandaka	M1	Sensitive	4
		STR SMX GEN TET	2
	NT	Sensitive	1
		TET	1

*S*. Anatum	A1	STR SMX TET TMS	1
	A2	STR SMX TET TMS	1
		SMX TET TMS	1
	A3	Sensitive	1
		STR SMX TET TMS	1

*S*. Ohio	O1	STR	1
	NT	Sensitive	3

*S*. Tennessee	T1	SMX TMS	1
		TET TMS	1

*S*. California	C1	Sensitive	1

*S*. Heidelberg	H1	Sensitive	3
		STR	2
		STR SMX GEN	1
		AMP CEP AUG FOX	1

*S*. Jerusalem	J1	TET	1

*S*. Lille	L1	TET CEP CHL	1
	NT	TET CEP CHL	1
		CHL	1

*S*. Muenchen	U1	SMX	1

*^a^Phage typing was done only for *S*. Enteritidis and *S*. Typhimurium isolates. ( ) = phage type*.

*^b^NT = not typable by phage typing (column 1) or PFGE (column 2)*.

*^c^Resistance profiles to the following antibiotics: AMP, ampicillin; AUG, augmentin; FOX, cefoxitin; CEP, cephalothin; GEN, gentamicin; KAN, kanamycin, STR, streptomycin; AMI, amikacin; NAL, nalidixic acid; TET, tetracycline; SMX, sulfadimethoxine; TMS, trimethoprim/sulfamethoxazole; and CHL, chloramphenicol. Sensitive: susceptible to the 19 antibiotics tested*.

Of the three S. Typhimurium strain types (T1.1, T1.2, and T1.3) present on Farm One, there were three instances where two of these strain types were present with chicks on the broiler chicken farm (T1.1 and T1.2) and chicken carcasses derived from these flocks (Table 4). There were also three other situations where these same *S*. Typhimurium strain types were only isolated from the farm environment and then chicken carcasses at processing. The only antibiotic resistant *S*. Typhimurium strain types found on chicken carcasses matched with those isolated from chicks at farm placement indicating that resistant *S*. Typhimurium strains were likely vertically transferred from the breeder flock.

**Table 4:** Temporal and spatial distribution of resident antibiotic susceptible and resistant *S*. Typhimurium strain types during the production of seven consecutive commercial broiler flocks.

Flock^a^	*S*. Typhimurium PFGE type	Antimicrobial resistance phenotype^b^	Location^c^		
			Hatchery	House	Carcass
1	T1.1	Sensitive		2	
		STR		2	
		STR SMX TET TMS		1	
		STR SMX GEN TET TMS		2	

2	T1.1	Sensitive		5	
	T1.2	Sensitive	3	16	
		STR	2	1	1
		STR SMX GEN		2	
		STR SMX GEN TET		1	
	T1.3	Sensitive		4	
		STR SMX GEN AMP		1	

3	T1.1	Sensitive	1	4	1
		STR	1	3	5
		STR SMX		1	
	T1.2	Sensitive		4	
		STR		1	
	T1.3	Sensitive		1	

4	T1.1	Sensitive		3	14
		STR			1

5	T1.1	Sensitive		8	1
		STR		2	
	T1.2	Sensitive		4	1
		STR		5	

6	T1.1	Sensitive		2	
		STR		2	
	T1.2	Sensitive		2	
		STR		9	
		STR SMX CEP		1	
		MDR^d^		1	

7	T1.1	Sensitive		7	
		STR SMX GEN TET		3	
	T1.2	Sensitive		15	
		STR		2	
		STR SMX GEN		3	
		STR SMX GEN TET		2	

*^a^Poultry Farm One*.

*^b^Resistance profiles to the following antibiotics: AMP, ampicillin; AUG, augmentin; FOX, cefoxitin; CEP, cephalothin; GEN, gentamicin; KAN, kanamycin, STR, streptomycin; AMI, amikacin; NAL, nalidixic acid; TET, tetracycline; SMX, sulfadimethoxine; TMS, trimethoprim/sulfamethoxazole; and CHL, chloramphenicol. Sensitive: susceptible to the 19 antibiotics tested*.

*^c^Number of Salmonella isolates belonging to said strain type and antimicrobial resistance phenotype*.

*^d^Multidrug resistance (MDR) to antibiotics: STR, SMX, FOX, AMI, AXO, NAL, TIO, and APR*.

## Discussion

The antibiotic susceptibility and profiles of *Salmonella* isolated from two poultry farms mirrored antimicrobial resistance data reported in other studies. The majority (51.6%) of *Salmonella* isolates, from a 2001 NARMS survey, were also pan-susceptible. The most commonly identified resistances were to the antibiotics tetracycline (26.7%), streptomycin (23.7%), sulfadimethoxine (9.1%), gentamicin (6.3%), and ampicillin (15.1%) (26). A 2002 NARMS retail survey also reported that *Salmonella* isolated from chicken meat were largely pan-susceptible (66.6%), with the most prevalent resistance observed for sulfadimethoxine (18.7%), streptomycin (32.3%), gentamicin (3.4%), ampicillin (5.1%), trimethoprim/sulfamethoxazole (1.7%), and tetracycline (34.3%) (4). The 2003 NARMS retail meats survey, contemporary with the sampling times of this study, reported 47% of *Salmonella* isolates as pan-susceptible; with resistances observed for tetracycline (27.4%), streptomycin (26.2%), sulfadimethoxine (14.3%), gentamicin (6.0%), and ampicillin (33.3%). In the most recent NARMS retail meats survey (2015), half of the poultry *Salmonella* isolates were pan-susceptible to a panel of 12 antibiotics. *Salmonella* isolated from retail meats, in this survey, were resistant to tetracycline (37.3%), streptomycin (37.3%), sulfadimethoxine (8.5%), gentamicin (5.1%), and ampicillin (8.5%) (27).

There was a diversity of antimicrobial resistance phenotypes identified among our poultry *Salmonella* isolates. Despite this diversity, the antimicrobial resistance phenotype: streptomycin and sulfadimethoxine resistance alone or with other antibiotics was commonly encountered in *Salmonella* isolated from the commercial poultry farms. The genes conferring resistance to these antimicrobials are frequently found residing on mobile genetic elements which are responsible for the wide-spread dissemination of antimicrobial resistance in nature. The transposon Tn*21* contains the mercury resistance gene *merA*; streptomycin resistance gene *aadA1*; and sulfadimethoxine resistance gene *sul1* (28). This transposon is often responsible for dissemination of mercury and antimicrobial resistance in nature (28) and is prevalent in poultry *Salmonella* and *E. coli* (23). While we observed linkage between the resistance genes *merA* and *aadA* and streptomycin/sulfadimethoxine resistance, only 17.72% of streptomycin-resistant *Salmonella* had *aadA1*, indicating that other antimicrobial resistance gene(s) are responsible for streptomycin resistance and further illustrates the diversity underlying antimicrobial resistance phenotypes observed in these isolates.

Despite the high prevalence of Tn*21* in these poultry isolates, antimicrobial resistance phenotypes were not uniformly distributed among *Salmonella* serovars within as well as between the two commercial broiler chicken farms. Certain *Salmonella* serovars differed in their antibiotic susceptibility patterns. *Salmonella* Enteritidis tended to be pan-susceptible while *S*. Typhimurium exhibited a diversity of antimicrobial resistance phenotypes. Similar trends have been observed for these *Salmonella* serovars reported in NARMS retail meats (2003, 2015) and HACCP (2003, 2014) surveys (27). Even within *S*. Typhimurium, there were differences in antibiotic susceptibilities among strain types. The *S*. Typhimurium PFGE subtype T1.1 from Farm One (21) was identified as phage type (PT) 193, a PT commonly associated with illnesses in humans (29–39). This *Salmonella* PT has also been isolated from cattle (38, 40, 41), poultry (31, 42), pigs (31, 40), and dogs (40). Like *S*. Typhimurium DT104, PT 193 isolates generally exhibit resistance to three or more antibiotics, but resistance phenotypes reported have been variable (40, 43). The majority (68.0%) of our *S*. Typhimurium PT DT193 isolates from Farm One were pan-susceptible, with 32% possessing the following resistance phenotypes to: streptomycin alone; streptomycin, sulfadimethoxine, tetracycline, and trimethoprim/sulfamethoxazole; streptomycin, sulfadimethoxine, gentamicin, tetracycline, and trimethoprim/sulfamethoxazole. The other *S*. Typhimurium PFGE types, T1.2 and T1.3, were identified, respectively, as PTs DT107 and U302 (21). The *S*. Typhimurium PTs DT107 and DT193 from this study appear to be genetically related as determined by PFGE (44). Close genetic-relatedness as determined by PFGE among different *S*. Typhimurium and *S*. Enteritidis PTs has been reported by others (45, 46). The *S*. Typhimurium DT107 isolates were similar to the *S*. Typhimurium DT193 isolates, in that the majority were pan-susceptible (59.6%), with the most prevalent antimicrobial resistance phenotype being resistance to streptomycin only (25.4%).

Poultry litter contains a large reservoir of antimicrobial resistance genes. We had shown in a previous study that many of these antimicrobial resistance genes are shared among diverse bacterial species in poultry litter (ex. *aadA1* in *Corynebacterium* and *Salmonella*) (20). Therefore, the potential exists for environmental transfer of antimicrobial resistance to *Salmonella* and subsequent horizontal transmission of emergent resistant *Salmonella* strains to poultry in this environment. Of the eight antibiotic resistant phenotypes solely present in *S*. Typhimurium isolated from the farm environment, none were identified in *S*. Typhimurium isolated from processed chicken carcasses. This finding suggests that despite the diversity of antibiotic-resistant *S*. Typhimurium resident in the broiler house environment, none of these antibiotic resistant strains were being transmitted through the processing plant to the poultry carcass. Only those antibiotic-resistant strain types present with the chicks at placement remained on birds at processing. Therefore, our data support the importance of vertical transmission routes in the dissemination of antibiotic-resistant *Salmonella* through the food chain.

## Conclusion

Therapeutic tetracycline antibiotic usage was not a significant predictor of emergent antimicrobial resistance in *Salmonella*. This result is not surprising, considering that the all-in, all-out production method used in the commercial poultry industry is designed to break disease cycles and should minimize antimicrobial resistance development, as long as pathogen persistence from flock-to-flock is prevented (47). However, the reservoir for antimicrobial resistance remains within the farm environment. Additional measures involving litter management and pest control may be needed to prevent future emergence of antimicrobial resistance zoonotic bacteria on treated farms. In addition, the prevalence of streptomycin resistance in poultry *Salmonella* was surprisingly high considering that streptomycin is rarely used in poultry production medicine and to our knowledge had not been used at these farms. This is most likely due to linkage of streptomycin resistance gene(s) with other resistance genes, or competitively advantageous genes (bacteriocins, siderophores, etc.); or its integration into the chromosome that has maintained streptomycin resistance in *Salmonella*, even in the absence of antibiotic selection (19). However, gentamicin is commonly used with *in ovo* poultry vaccines as a prophylaxis against peritonitis in chicks and therefore may explain, in part, the level of resistance to this antibiotic in *Salmonella*. The physical linkage of resistance genes associated with gentamicin with streptomycin resistance may also explain the persistence of streptomycin resistance in the absence of usage (19). As gentamicin was used by both poultry companies, it is uncertain whether gentamicin resistance in *Salmonella* will persist with time. The other antibiotics used by the poultry farms in this study, bacitracin and virginiamycin, are used to control *C. perfringens* infections in poultry. While these antibiotics do not affect *Salmonella* or other Gram-negative enterics, they do have an impact in the Gram positive, intestinal microbiota of chickens (48). It is currently not known how changes to the chicken intestinal microbiota, in response to bacitracin and virginiamycin, affect *Salmonella* prevalence, abundance, or antibiotic resistance patterns.

Vertical transmission from the breeder flock, rather than horizontal transmission from the environment, appears to play a significant role in carcass contamination with antibiotic-resistant *Salmonella*. If antibiotic usage is involved in the emergence and spread of antibiotic-resistant *Salmonella* to chicken meat, it may exist at the breeder, not broiler level of poultry production. One way to block transmission of antimicrobial-resistant *Salmonella* would be to apply an intervention such as competitive exclusion or vaccination at the breeder level (49, 50). The poultry integrator for Poultry Farm One has recently instituted a company-wide *Salmonella* vaccination program at the broiler-breeder level. It will be interesting to see if this mitigation strategy has significantly changed antimicrobial resistance profiles of *Salmonella* isolated from broiler chicken farms and poultry charges, especially on Poultry Farm One.

## Author Contributions

JM, CH, DW, and ML contributed to the conception and design of this study. KL and SA were responsible for the acquisition of data analyzed in this study. JM, KL, and DW were involved in the analysis and interpretation associated with this work. KL was responsible for writing the first draft. All the authors were involved in manuscript revisions and final approval of the version to be published.

## Conflict of Interest Statement

None of the authors received any financial gain or applied for patents associated with the work described in this article. The reviewer, TP, and handling editor declared their shared affiliation, and the handling editor states that the process nevertheless met the standards of a fair and objective review.
